# Endoscopic Ultrasound-Guided Pancreaticogastrostomy for Pancreatic Duct Stricture in a Patient With Ansa Pancreatica

**DOI:** 10.14309/crj.0000000000001876

**Published:** 2025-11-17

**Authors:** Abdullah A. Abbasi, Saurabh Chandan, Sagar Pathak, Dennis Yang, Kambiz Kadkhodayan, Muhammad Hasan, Mustafa A. Arain

**Affiliations:** 1 Center of Interventional Endoscopy (CIE), Advent Health Orlando, Orlando, FL; 2 University of Iowa Health Care Medical Center, Iowa City, IA; 3 Houston Methodist Gastroenterology Assosiates, Houston Methodist West Hospital, Houston, TX; 4 Division of Gastroenterology and Hepatology, Loma Linda University, Loma Linda, CA

**Keywords:** acute pancreatitis, endoscopic ultrasound, therapeutic endoscopy, EUS-guided pancreaticogastrostomy

## Abstract

Necrotizing pancreatitis can lead to challenging complications such as pancreatic duct stricture and pancreatic duct disruption. We report a 59-year-old woman with pancreatic ductal stricture causing pancreatic duct leak, initially managed with endoscopic ultrasound-guided pancreaticogastrostomy (PG) after failed transpapillary access due to ansa pancreatica. At follow-up, the PG stent enabled minor papilla cannulation and successful pancreatic duct stenting. The PG stent was removed, and durable transpapillary drainage was achieved. The patient remained asymptomatic with resolution of ductal and peripancreatic abnormalities. This case highlights a minimally invasive, stepwise endoscopic approach for complex ductal anatomy in necrotizing pancreatitis, avoiding surgical intervention.

## INTRODUCTION

Acute pancreatitis has significant healthcare implications in the United States, with over 130,000 reported cases every year.^1^ Roughly, 20%–30% of these can progress to necrotizing pancreatitis, which is associated with significant morbidity and mortality.^2^ The clinical course of necrotizing pancreatitis can be complex, often requiring multidisciplinary approach to management. Pancreatic duct stricture leading to peripancreatic fluid collections is among the more challenging complications of necrotizing pancreatitis.^3^ The Dutch Pancreatitis Study Group has proposed useful management algorithm that can assist in the care of these complex patients.^4^ The traditional approach of early surgery was associated with high mortality and has therefore been replaced by various endoscopic treatment options, now considered as first-line therapy.^5^

Endoscopic management of such cases requires high level of skill and expertise. We present an interest case of a patient with acute pancreatitis complicated by high-grade pancreatic duct stenosis causing peripancreatic fluid collections, requiring multiple endoscopic interventions. We discuss the unique anatomical challenges encountered in this case and their management.

## CASE REPORT

**Figure 1.**
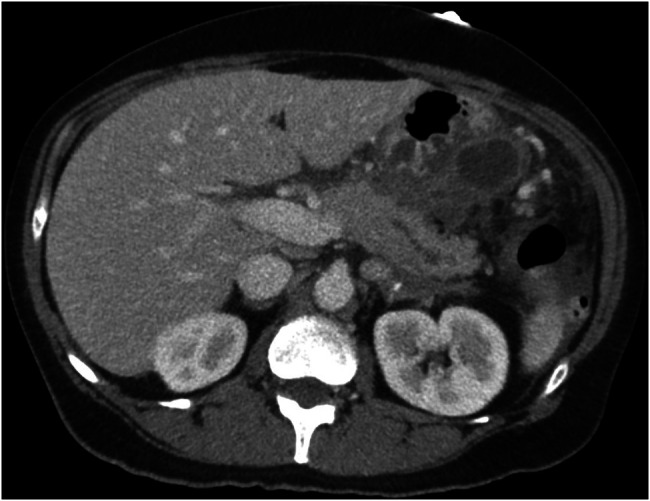
Computed tomography scan on index presentation consistent with peripancreatic fluid collection, dilated pancreatic duct, and changes consistent with pancreatitis.

A 59-year-old female patient with a history of hypothyroidism presented to our hospital with sudden onset of upper abdominal pain. Physical examination was positive for generalized abdominal pain. Laboratory tests showed a white blood cell count of 11.9 × 10^9^/L (normal range: 4.5–11.0 × 10^9^/L) and an elevated lipase of 4,425 U/L (normal range: 30–150 U/L). Her hepatic panel was normal, and on transabdominal ultrasound, she had multiple gallstones and a normal caliber common bile duct. A contrast-enhanced computed tomography scan demonstrated cholelithiasis and acute necrotizing pancreatitis in Figure 1.

The patient was managed conservatively with analgesia and intravenous hydration. She was discharged after 1 week of hospital stay. She had follow-up in the surgery clinic and underwent a laparoscopic cholecystectomy 6 weeks later for symptomatic cholelithiasis and suspected gallstone pancreatitis. However, she continued to experience persistent upper abdominal pain on follow-up in the pancreas clinic.

**Figure 2.**
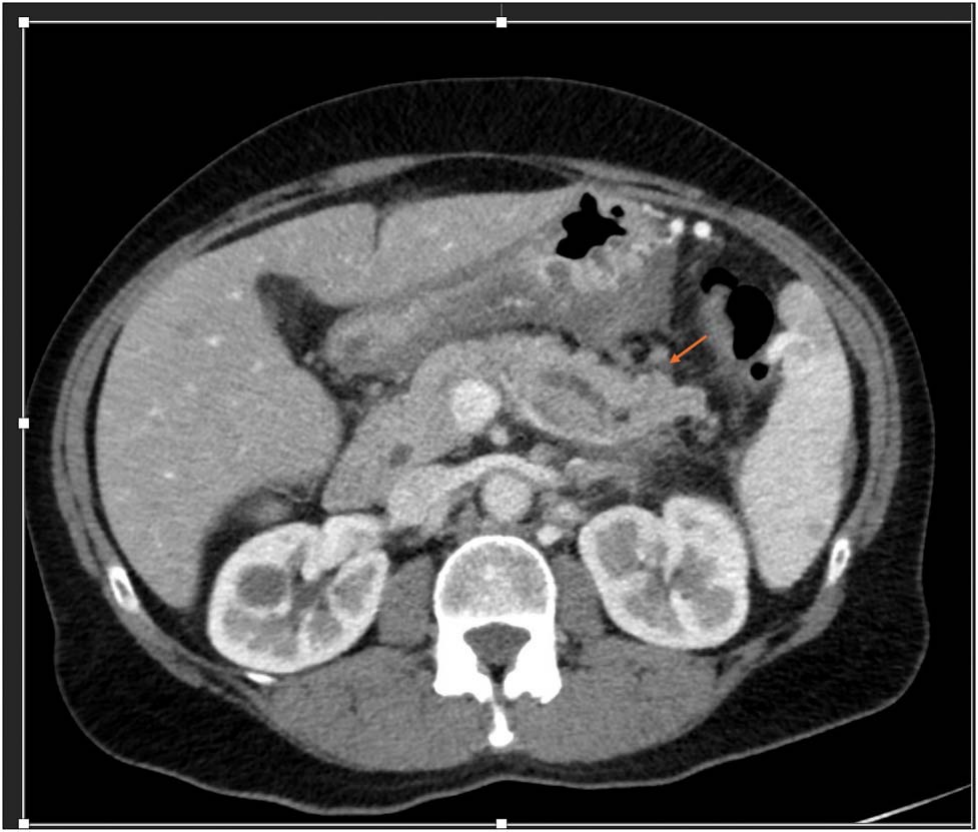
Repeat imaging 3 months after index presentation showed persistently dilated pancreatic duct in the distal body and tail of pancreas.

Repeat imaging 3 months after the initial presentation revealed an evolving peripancreatic fluid collection with suspicion of a high-grade stricture in the distal body of pancreas, leading to a peripancreatic fluid collection due to pancreatic duct disruption. The pancreatic duct appeared dilated upstream toward the tail of pancreas in Figure 2. The peripancreatic collection was poorly defined and small and was not deemed suitable for transgastric drainage.

**Figure 3.**
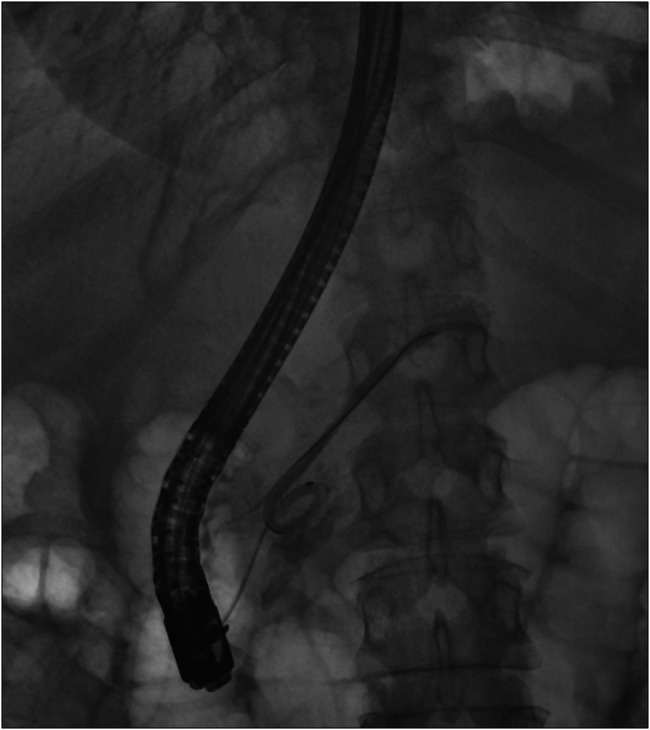
Pancreatogram on endoscopic retrograde cholangiopancreatography showed an ansa loop and dilated dorsal pancreatic duct. There is an area of stenosis in the distal body of pancreas which limits the passage of wire in the tail of pancreas.

After discussing various treatment options, endoscopic retrograde cholangiopancreatography (ERCP) and pancreatic duct stenting were planned. Initial successful ventral duct cannulation was achieved. The pancreatogram revealed an ansa loop (ansa pancreatica) of the main pancreatic duct. Although a 0.025 inch glide wire was passed beyond the loop, it significantly limited the advancement of other accessories. An abrupt cutoff of the distal pancreatic duct raised concerns for a severe pancreatic duct stenosis in Figure 3. In addition, the pancreatogram also showed a dilated branch of dorsal pancreas suggestive of pancreatic duct divisum; however, the minor papilla cannulation was unsuccessful due to severe stenosis. Cholangiogram showed inflammatory biliary stricture managed by placement of metal stent.

**Figure 4.**
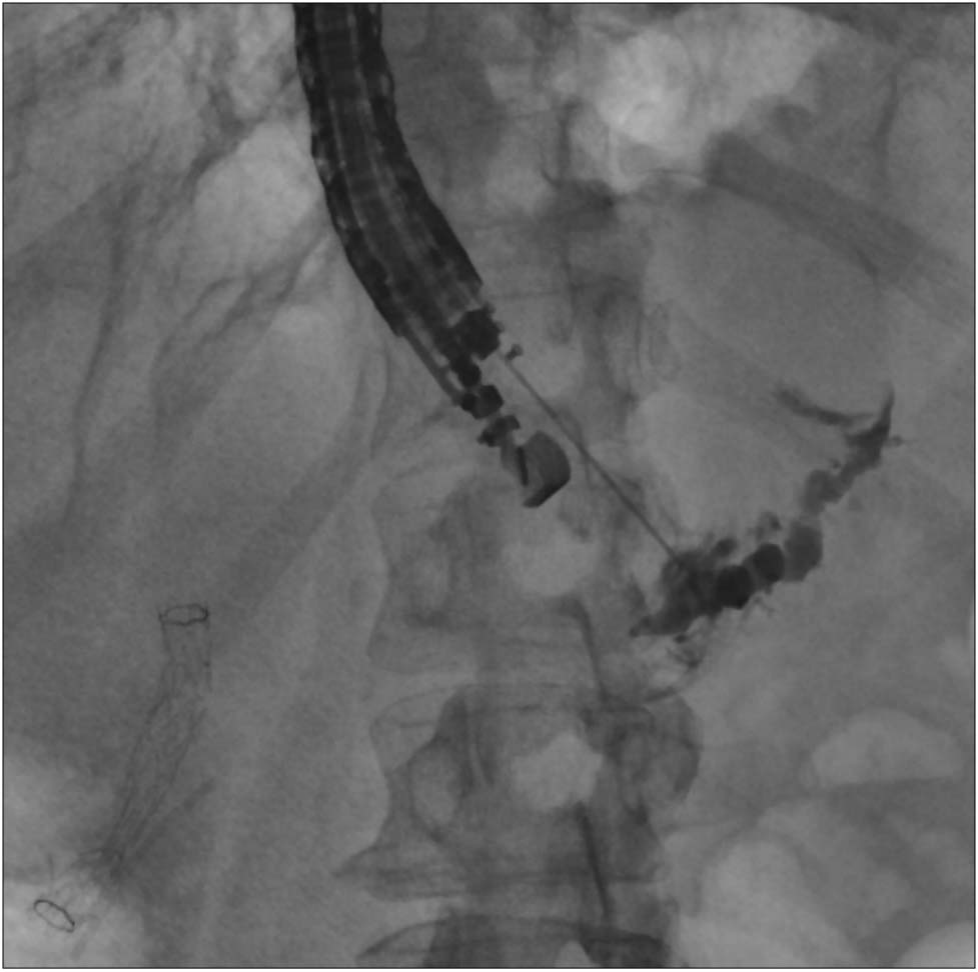
Endoscopic ultrasound-guided pancreaticogastrostomy was created distal to the stricture. Pancreatogram confirms area of contrast extravasation and dilated pancreatic duct in the distal body and tail of pancreas.

**Figure 5.**
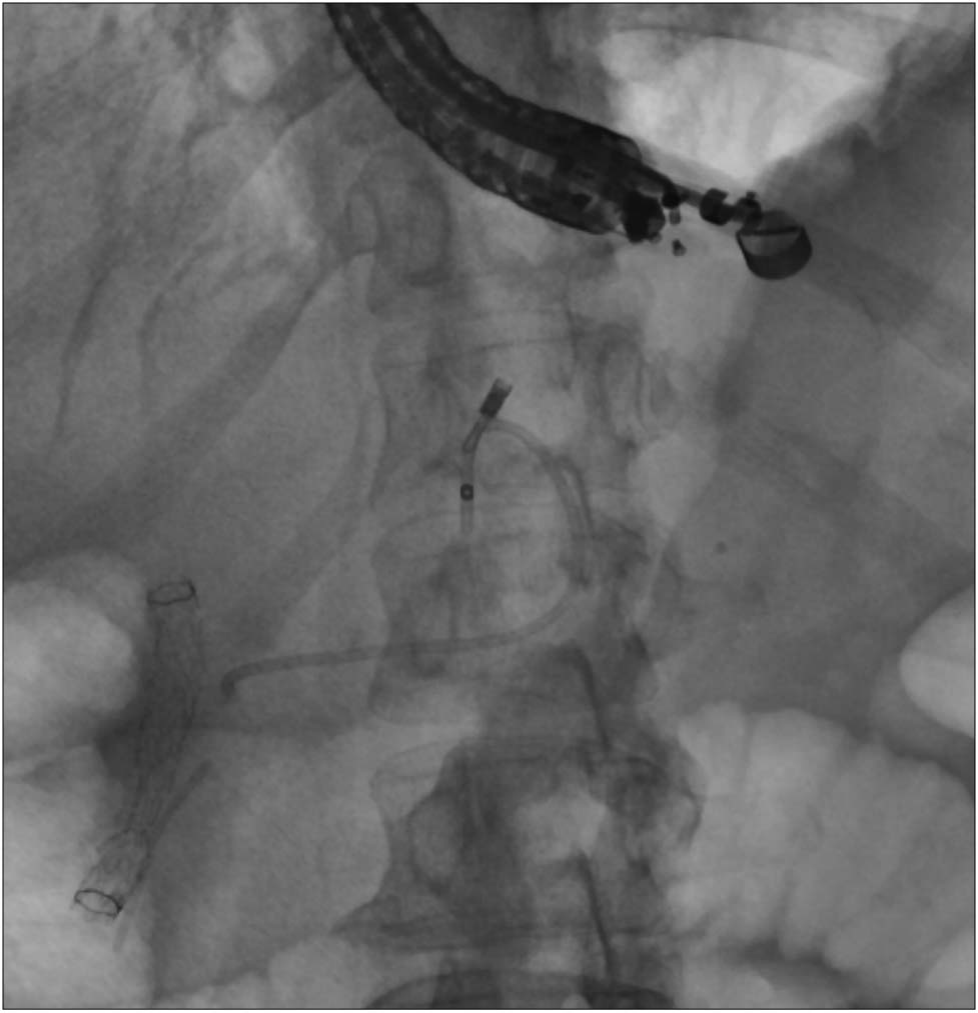
Successful placement of pancreaticogastrostomy stent which was secured with a clip on gastric side. Recently placed biliary stent and transpapillary pancreatic stent up to the neck of pancreas are visible too.

Subsequently, endoscopic ultrasound (EUS) was performed with the aim of draining the obstructed and leaking duct in the tail of the pancreas. The pancreatic duct was identified, and a 22 G needle was used to puncture the upstream from the area of suspected stricture in Figure 4. Pancreatogram confirmed contrast extravasation from the tail of pancreas, consisted with ductal disruption in the tail of pancreas. A guidewire was advanced through the stricture, toward the head of pancreas, and cystotome was used for successful creation of the pancreaticogastrostomy (PG) tract. One 5 Fr by 9 cm plastic stent was placed through PG tract toward the head of pancreas in Figure 5.

**Figure 6.**
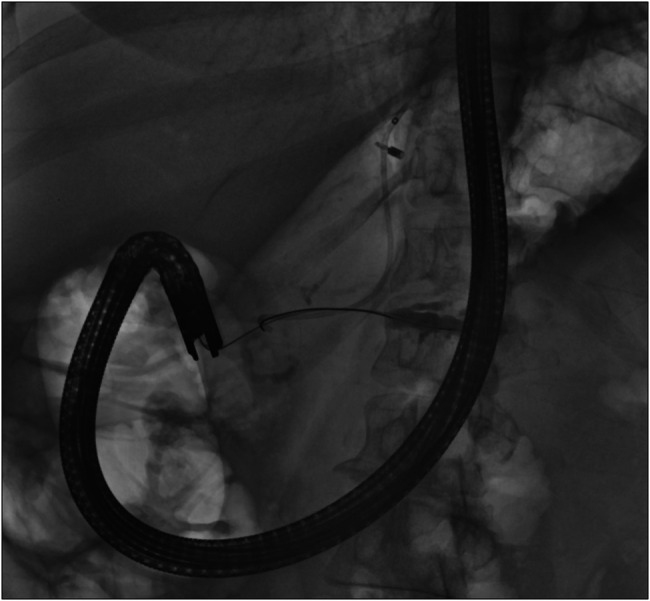
Minor papilla was cannulation in the long scope position, and wire was negotiated till the tail of pancreas. Pancreatogram showed dilated pancreatic duct in the distal body and tail.

**Figure 7.**
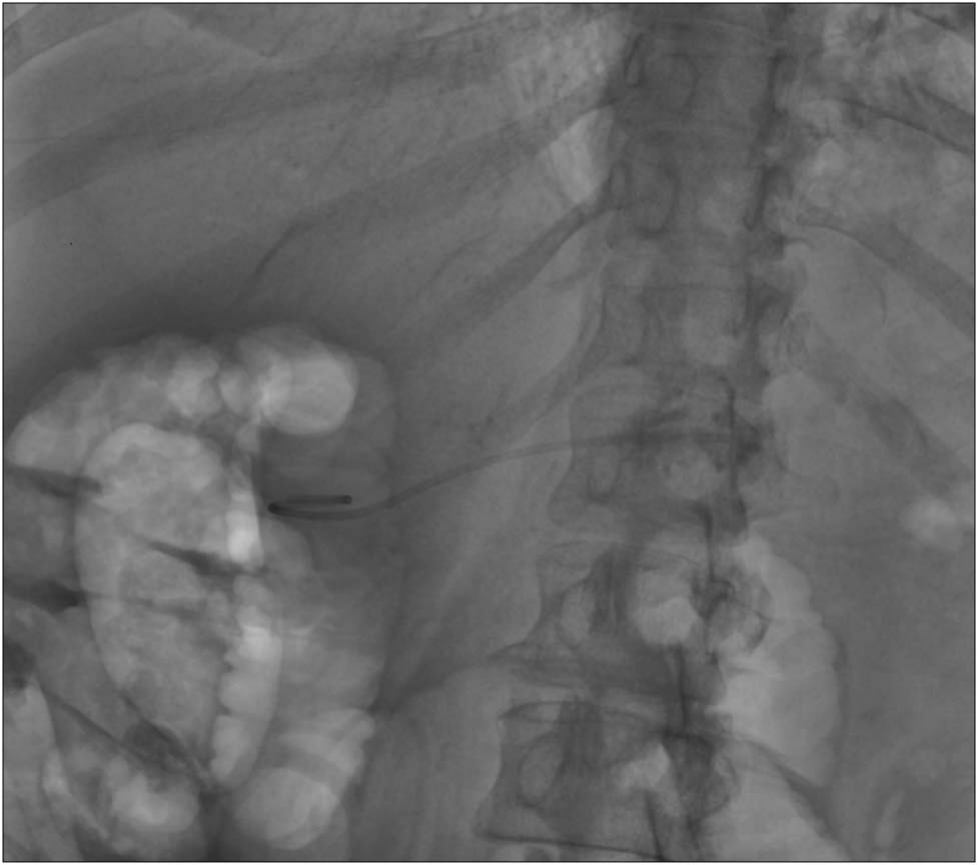
Eventual placement of transpancreatic plastic stent till the tail of pancreas with removal of pancreaticogastrostomy stent.

At the 2-month follow-up, patient's symptoms had improved significantly. Repeat computed tomography imaging showed marked improved in the pancreatic fluid collections. During follow-up ERCP, minor papilla cannulation was successful. The previously placed pancreatogastrostomy stent was used as a guide to achieve deep pancreatic duct cannulation bypassing the limitation of ansa loop in Figure 6. This was followed by placement of 5 Fr × 10 cm pancreatic plastic stent across the minor papilla, and PG stent was removed in Figure 7. The patient's pain symptoms improved significantly following the procedure, and she was discharged home 2 days later.

On follow-up, the patient was asymptomatic. Her follow-up imaging and ERCP showed almost complete resolution of peripancreatic fluid collection with no subsequent extravasation of contrast and resolution of pancreatic duct stenosis.

## DISCUSSION

Since the first reported case series in 2002, EUS-guided PG has become an established treatment modality for managing pancreatic duct stenosis or disconnected pancreatic duct syndrome.^6,7^ It serves as an alternate to more aggressive surgical options, including resection of upstream gland with or without islet cell transplant or creating a surgical pancreaticojejunostomy.^6,8^ Also, surgery in these patients in associated with high mortality and morbidity along with long-term exocrine and endocrine insufficiency.^4,5^ It should be noted that EUS-guided PG is typically reserved for patients with pancreatic duct obstruction in the setting of chronic pancreatitis or a chronically disconnected pancreatic duct rather than pancreatic duct injury in the setting of acute pancreatitis.^6^

The traditional approach in such cases aims for transpapillary access by ERCP, enabling both pancreatic duct stenting and dilatation of pancreatic duct strictures. Alternatively, EUS-guided creation of a pancreaticoenteric tract allows for long-term stents and internal drainage.^5^ However, this approach is limited by lack of robust long-term outcome data and carries risk of stent migration.

Our case is unique in several aspects. Following the patient's initial presentation with recurrent pancreatic fluid collection, transpapillary approach through the ventral duct was limited by the presence of ansa loop. In addition, minor papilla cannulation was unsuccessful due to papillary stenosis. This challenge was managed by the creation of pancreaticogastrostomy. Given the uncertain long-term outcome of indwelling pancreaticoenteric stents, our goal was to re-establish transpapillary access for definitive management of pancreatic duct stenosis and eventually give her a stent-free period. This was successful during follow-up ERCP through minor papilla, enabling long-term pancreatic duct stenting and resolution of the ductal disconnection.

This case highlights a unique and minimally invasive endoscopic approach for managing ductal complications after necrotizing complications. Our strategy successfully addressed anatomical challenges and re-established transpapillary pancreatic duct access. A multidisciplinary, patient-tailored approach remains essential to achieving optimal outcomes in complex pancreatic ductal disease.

## DISCLOSURES

Author contributions: M. Arain and A. Abbasi was involved in the conception and design of the study. M. Arain and A. Abbasi were involved in the drafting of the manuscript. All authors were involved in the critical appraisal and final approval of the manuscript. M. Arain and A. Abbasi are the article guarantors and take full responsibility for the integrity of the work as a whole, including the accuracy of the data, the decision to publish and ensuring that all authors meet the criteria for authorship.

Financial disclosure: Muhammad K. Hasan: Consultant for Boston Scientific, Olympus, Neptune Medical and Microtech. Dennis Yang: Consultant for Boston Scientific, Fujifilm, Olympus, Medtronic, Microtech, 3D-Matrix, Neptune Medical, ERBE and Aspero Medical. Mustafa Arain: Consultant for Boston, Olympus and Cook Medical. All other authors have nothing to disclose.

Informed consent was obtained for this case report.

## ABBREVIATIONS:

EUS: endoscopic ultrasound
PG: pancreaticogastrostomy
ERCP: endoscopic retrograde cholangiopancreatography
